# Automated Quantitative Analysis of Anterior Segment Inflammation Using Swept-Source Anterior Segment Optical Coherence Tomography: A Pilot Study

**DOI:** 10.3390/diagnostics12112703

**Published:** 2022-11-05

**Authors:** Hiroshi Keino, Takuto Aman, Ryota Furuya, Makiko Nakayama, Annabelle A. Okada, Wataru Sunayama, Yuji Hatanaka

**Affiliations:** 1 Department of Ophthalmology, Kyorin University School of Medicine, 6-20-2 Shinkawa, Tokyo 181-8611, Japan; 2 Faculty of Science and Engineering, Oita University, 700 Dannoharu, Oita 870-1192, Japan; 3 Faculty of Engineering, The University of Shiga Prefecture, 2500 Hassaka, Hikone 522-8533, Japan

**Keywords:** uveitis, anterior chamber inflammation, optical coherence tomography (OCT), image analysis, Standardization of Uveitis Nomenclature (SUN)

## Abstract

Background: The aim of this study is to develop an automated evaluation of anterior chamber (AC) cells in uveitis using anterior segment (AS) optical coherence tomography (OCT) images. Methods: We analyzed AS swept-source (SS)-OCT (CASIA 2) images of 31 patients (51 eyes) with uveitis using image analysis software (Python). An automated algorithm was developed to detect cellular spots corresponding to hyper-reflective spots in the AC, and the correlation with Standardization of Uveitis Nomenclature (SUN) grading AC cells score was evaluated. The approximated AC grading value was calculated based on the logarithmic approximation curve between the number of cellular spots and the SUN grading score. Results: Among 51 eyes, cellular spots were automatically segmented in 48 eyes, whereas three eyes (all SUN grading AC cells score: 4+) with severe fibrin formation in the AC were removed by the automated algorithm. The AC cellular spots increased with an increasing SUN grading score (*p* < 0.001). The 48 eyes were split into training data (26 eyes) and test data (22 eyes). There was a significant correlation between the SUN grading score and the number of cellular spots in 26 eyes (rho: 0.843, *p* < 0.001). There was a significant correlation between the SUN grading score and the approximated grading value of 22 eyes based on the logarithmic approximation curve (rho: 0.774, *p* < 0.001). Leave-one-out cross-validation analysis demonstrated a significant correlation between the SUN grading score and the approximated grading value of 48 eyes (rho: 0.748, *p* < 0.001). Conclusions: This automated anterior AC cell analysis using AS SS-OCT showed a significant correlation with clinical SUN grading scores and provided SUN AC grading values as a continuous variable. Our findings suggest that automated grading of AC cells could improve the accuracy of a quantitative assessment of AC inflammation using AS-OCT images and allow the objective and rapid evaluation of anterior segment inflammation in uveitis. Further investigations on a large scale are required to validate this quantitative measurement of anterior segment inflammation in uveitic eyes.

## 1. Introduction

Uveitis remains a significant cause of visual loss [1,2,3]. Uveitis is classified into four types based on its anatomical location: anterior uveitis, intermediate uveitis, posterior uveitis, and panuveitis [4]. Anterior uveitis is the most common form of uveitis in Western countries [5]. The major clinical findings of anterior uveitis are the presence of cells and flare in the anterior chamber. Anterior uveitis is generally managed by topical treatment, including corticosteroid eye drops. Since long-standing inflammation in the anterior chamber can lead to vision-threatening ocular complications such as cataracts and glaucoma, the control of intraocular inflammation is important for preventing the development of ocular complications.

Slit-lamp examination is the standard method to observe the anterior chamber in the clinical practice of uveitis, and the precise assessment of intraocular inflammation in the anterior segment is critical for diagnosis, the decision of therapy, and monitoring the response to treatment. Currently, the Standardization of Uveitis Nomenclature (SUN) grading system is widely used to evaluate the disease activity of uveitis, and anterior segment inflammatory cells are graded clinically from 0 to 4+ based on SUN guidelines using the slit-lamp examination [4]. However, the SUN scoring system is highly subjective and has some limitations, including variations in grading between examiners, unequal intervals between grades, and low sensitivity to small fluctuations on the severity scale [4,6,7,8]. In addition, the ability to count cells in the anterior chamber depends on the illumination level of the slit-beam [9]. Although the quantitative assessment of aqueous flare is possible using a laser flare photometer [10], there are no commercially available devices to count anterior chamber cells automatically. Therefore, objective and quantitative methods to grade inflammation in the anterior chamber are required for the management of patients with uveitis and clinical trials.

Anterior segment optical coherence tomography (AS-OCT) is a non-invasive imaging method for the qualitative and quantitative assessment of many anterior segment parameters. Recently, there has been accumulating evidence that AS-OCT, including spectral-domain OCT and swept-source OCT, allows for the detection of cells in the anterior chamber [11,12,13,14,15]. Although these studies have demonstrated the usefulness of AS-OCT for objective and quantitative analysis of anterior chamber inflammation, studies of the automated detection of cells in patients with uveitis using AS-OCT are limited [14,16]. Additionally, there are no studies regarding the automated measurement of SUN AC grading values as a continuous variable using AS-OCT imaging. In the current study, we analyzed anterior chamber cells in patients with uveitis using swept-source OCT images and developed an automated program for the measurement of cellular spots using imaging software. Our automated analysis of anterior chamber cells using AS-OCT images demonstrated that there was a significant correlation between clinical SUN grading scores and approximated anterior chamber grading values with the logarithmic approximation curve between the SUN grading scale and the number of cellular spots in the anterior chamber. The present study suggests that automated grading of anterior chamber cells could improve the accuracy of a quantitative assessment of anterior chamber inflammation using AS-OCT images and facilitate the standardization of clinical practice of uveitis.

## 2. Materials and Methods

### 2.1. Subjects and Clinical Examination

This study was conducted in accordance with the tenets of the Declaration of Helsinki, and the ambidirectional study was approved by the Kyorin University Hospital Research Ethics Committee (protocol code 800 and 11 June 2021). The retrospective portion of the study was conducted from January 2020 to May 2021, and the prospective portion was conducted from June 2021 to January 2022. Written informed consent was waived for the retrospective portion, while written informed consent was obtained from participants in the prospective portion. All of the patients (31 in number, 51 eyes) received a comprehensive ocular examination conducted by uveitis specialists (HK or MN). All of the patients were diagnosed with bilateral or unilateral uveitis involving the anterior segment (anterior uveitis or panuveitis). The grading of the anterior chamber cells was performed according to the SUN criteria [4] and was conducted before imaging by anterior segment OCT. Eyes having corneal disease with corneal opacification were excluded.

The diagnosis of specific uveitic disorders was based on a detailed clinical history, an extensive review of systems, a complete ophthalmologic examination, and uveitis-directed laboratory testing when deemed necessary [17]. Vogt-Koyanagi-Harada (VKH) disease was diagnosed using the Revised Diagnostic Criteria [18]. The diagnosis of acute retinal necrosis (ARN) was based on diagnostic criteria reported by the Japan ARN study group [19]. Ocular sarcoidosis was diagnosed using the International Workshop for Ocular Sarcoidosis revised criteria [20]. The diagnosis of Behçet’s disease was based on the Behçet’s Disease Research Committee of Japan criteria [21]. Varicella zoster virus (VZV) iridocyclitis was diagnosed by the presence of skin lesions characteristic of herpes zoster ophthalmicus. Unilateral granulomatous inflammation, increased intraocular pressure, and corneal edema or Descemet’s folds, as well as iris atrophy, were considered indicative of cytomegalovirus (CMV) infection, and polymerase chain reaction (PCR) testing for CMV in an aqueous humor was performed to confirm the diagnosis [22].

### 2.2. OCT Measurements

All uveitic eyes were imaged using the CASIA 2 (Tomey, Nagoya, Japan) swept source OCT by expert operators masked to the diagnosis and clinical grading result. CASIA 2 uses a longer wavelength of light (1310 nm) to image the anterior segment of the eye at 50,000 A-scans per second. The axial and lateral resolutions of this device are 10 mm and 30 mm, respectively, the lateral scan dimension is 16 mm, and the scan (axial) tissue depth is 13 mm. Four evenly distributed OCT images (every 45°) from radial scans (0°–180°) were obtained for each eye.

### 2.3. Image Analysis

**Figure 1:**
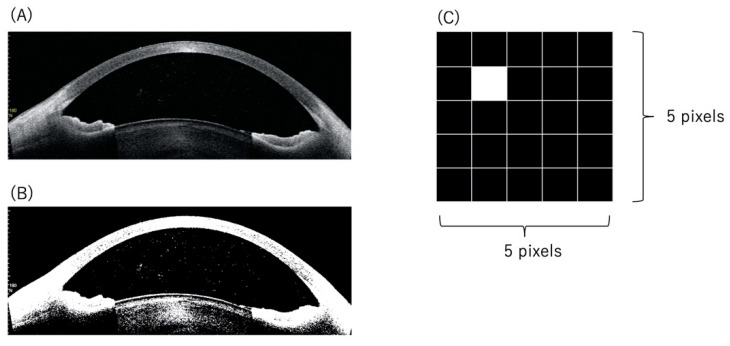
(**A**) Anterior segment OCT image in a representative case (SUN grading anterior chamber cells: 3+), (**B**) binarization of the OCT image, and (**C**) definition of cellular spots. (**A**) Hyperreflective spots were observed in the anterior chamber. (**B**) White spots corresponding to the hyperreflective spots were observed in the binarized image. (**C**) Anterior segment OCT images were divided into a single 5 × 5 pixel area. The 5 × 5 pixel areas were independent of each other. A raster scan was performed for each 5 × 5 pixel image in the anterior segment OCT image; and if a white area was detected in at least one pixel, it was judged to have the presence of inflammatory cellular spots, and the number of the cellular spots was then counted in the anterior chamber of each eye.

The 4 evenly distributed OCT images were analyzed. Image processing was performed with binarization and morphology transformation to reduce noise interference. The anterior chamber space was segmented by automated contour detection using the algorithm by Suzuki et al. included in Open CV [23]. The algorithm used in this study identified and quantified hyperreflective signals that exceeded a certain threshold reflectance value in the anterior chamber. It is difficult to detect cellular spots in OCT images of uveitic eyes with severe fibrin formation and increased protein content. Thus, if the number of white pixels exceeded 20% of 2 square regions with 1/8 width and height of an image in anterior chamber space, then these OCT images were automatically removed from the quantitative analysis. The anterior segment OCT images were divided into a 5 × 5 pixel area. The 5 × 5 pixel areas were independent of each other. To detect AC cells of various sizes and cell clumping, the detection of cellular spots was performed for each 5 × 5 pixel area in the anterior segment OCT image. Additionally, if a white area was found in at least one pixel, it was judged to have the presence of cellular spots. The number of cellular spots was counted in the anterior chamber of each eye (Figure 1). Image analysis was performed using Python with Open CV.

### 2.4. Evaluation of SUN Grading Using Automatic Segmentation of Cellular Spots in Anterior Chamber and Leave-One-Out Cross-Validation (LOOCV) Analysis

After measuring the number of cellular spots in the anterior chamber based on the image analysis described above, a logarithmic approximation curve was calculated using SUN grading in each eye and the number of cellular spots in each eye. The SUN grading score was calculated using the logarithmic approximation curve, and leave-one-out cross-validation analysis (LOOCV) was performed, leaving each one of the 48 eyes.

### 2.5. Statistical Analysis

Statistical analysis was performed using SPSS version 28. For the non-parametric test, the Kruskal–Wallis test was used, and the correlation analysis was performed using Spearman’s correlation analysis. A *p*-value less than 0.05 was considered statistically significant.

## 3. Results

### 3.1. Demographics

The demographic characteristics of the 31 patients (51 eyes) are summarized in Table 1. Twenty-one patients were men, and ten patients were women, all with ages ranging from 8 to 83 years (a mean of 55 years). There were four cases of Vogt-Koyanagi-Harada (VKH) disease, four cases of ocular sarcoidosis, two cases of Behçet’s disease, two cases of cytomegalovirus (CMV) anterior uveitis, one case of herpetic keratouveitis, one case of acute retinal necrosis (ARN), and 17 unclassified uveitis, respectively. 

### 3.2. Comparison of the Number of Cellular Spots in Anterior Chamber according to SUN Grading

**Figure 2:**
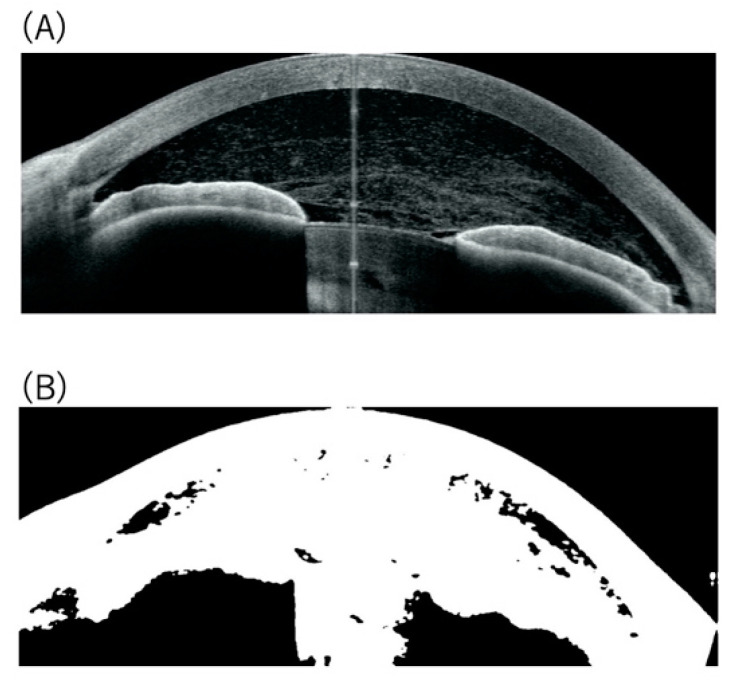
(**A**) Anterior segment OCT image in a representative case (SUN grading anterior chamber cells: 4), and (**B**) binarization of the OCT image. (**A**) Extensive hyperreflective spots due to severe fibrin formation and infiltration of inflammatory cells were observed in the anterior chamber. The fixation artefact observed as a vertical line in the middle of the anterior chamber in the AS-OCT was excluded using binarization and morphology transformation. (**B**) Extensive contiguous white spots corresponding to the hyperreflective spots were observed in the binarized image.

**Figure 3:**
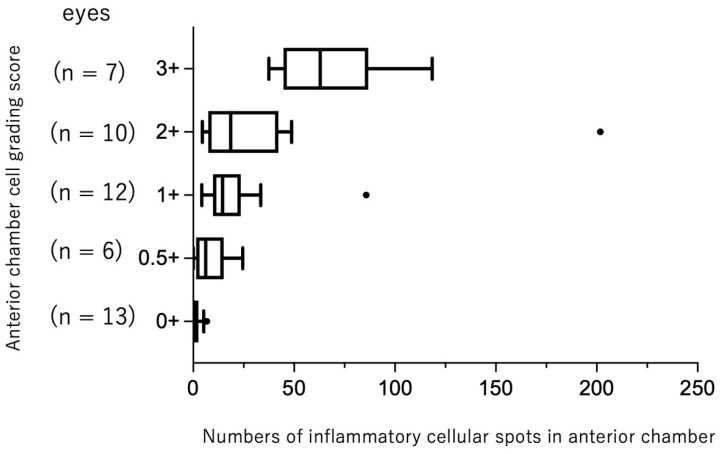
Numbers of cellular spots in anterior chamber according to SUN grading for anterior chamber cells. The number of cellular spots increased with SUN grading score (*p* < 0.001, Kruskal–Wallis test). Black dots represent outliers.

According to the SUN grading for the anterior chamber cells in each eye, 13 eyes had a grade of 0, six eyes had a grade of 0.5+, 12 eyes had a grade of 1+, 10 eyes had a grade of 2+, seven eyes had a grade of 3+, and three eyes had a grade of 4+. Among 51 eyes, cellular spots in the anterior chamber were segmented in 48 eyes, whereas three eyes with fibrin formation and intense flare were automatically removed due to the extensive contiguous white spots corresponding to severe fibrin formation and increased protein content (Figure 2). These three eyes had a grade of 4+ according to the SUN grading for anterior chamber cells. After excluding three eyes, 48 eyes were used for the automated segmentation of cellular spots in the anterior chamber. The cellular spots of 48 eyes, according to the SUN grading score, are shown in Figure 3. The cellular spots in the anterior chamber increased with an increasing SUN grading score (*p* < 0.001). 

### 3.3. Association between SUN Grading Score and the Number of Cellular Spots in Anterior Chamber

**Figure 4:**
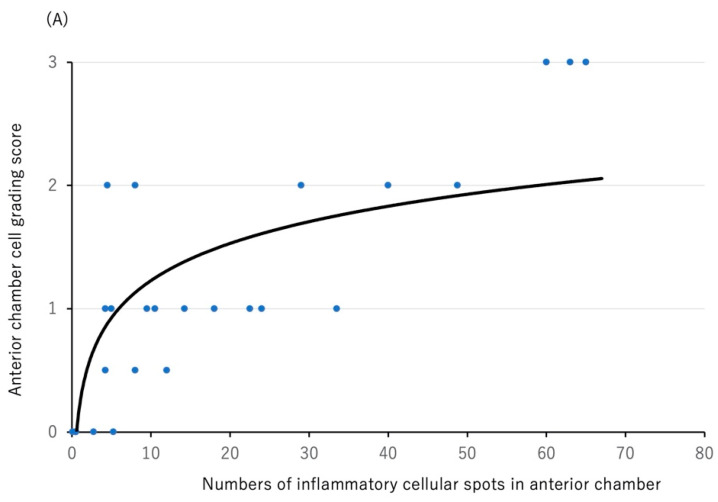

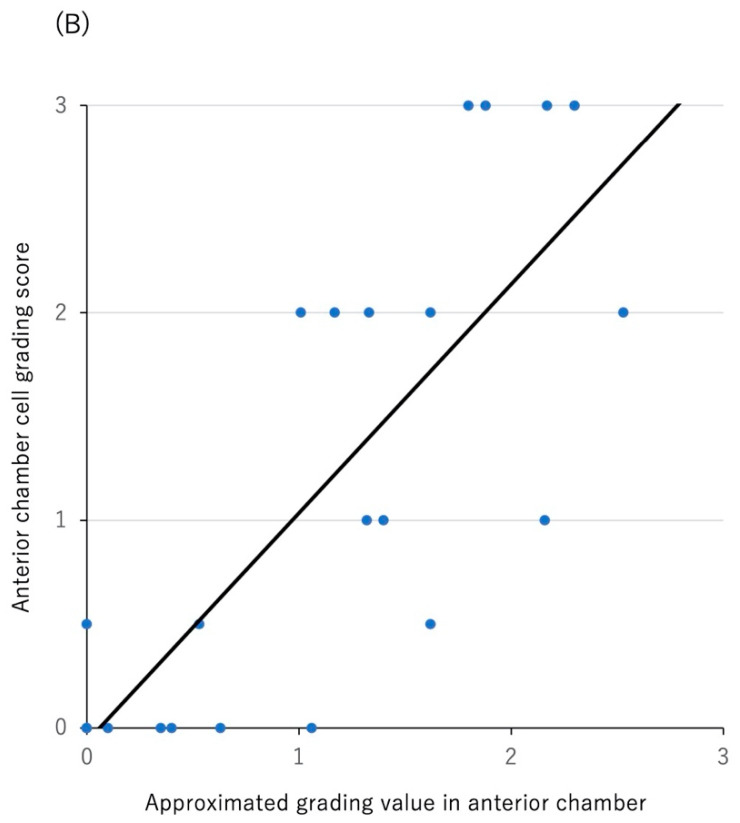
Scatter plot analysis between SUN grading score and number of cellular spots (**A**) and between the SUN grading score and the approximated grading value in each eye (**B**). (**A**) SUN grading score was significantly correlated with number of cellular spots (Rho: 0.843, *p* < 0.001, Spearman’s correlation analysis) and logarithmic approximation curve. (**B**) SUN grading score was significantly correlated with approximated grading value calculated with the logarithmic approximation curve in Figure 4A (Rho: 0.774, *p* < 0.001, Spearman’s correlation analysis) and an approximate straight line.

In the present study, 48 eyes were split into two groups: training data (26 eyes) and test data (22 eyes). Scatter plot analysis and calculation of a logarithmic approximation curve were performed using the 26 eyes as training data. There was a significant correlation between the SUN grading score and the number of cellular spots (rho: 0.843, *p* < 0.001) (Figure 4A). A logarithmic approximation curve was followed:

y = 0.4347ln(x) + 0.2276.

Next, the grading value was calculated with the remaining 22 eyes based on the logarithmic approximation curve as described above. As seen in Figure 4B, there was a significant correlation between the SUN grading score and the approximated grading value of each eye (rho: 0.774, *p* < 0.001). 

### 3.4. LOOCV Analysis

**Figure 5:**
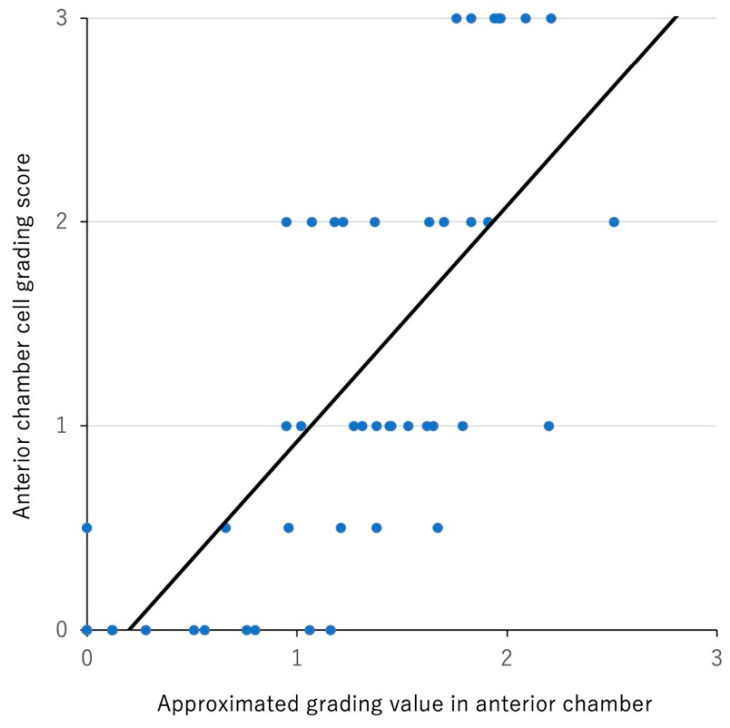
Scatter plot analysis between SUN grading score and approximated grading value in anterior chamber by LOOCV analysis. SUN grading score was significantly correlated with the approximated grading value in the anterior chamber by LOOCV analysis. (Rho: 0.748, *p* < 0.001, Spearman’s correlation analysis) and an approximate straight line.

Next, to evaluate how the results in this study could be generalized to a larger dataset, the LOOCV test was performed using the SUN grading score and the number of cellular spots in 48 eyes. As shown in Figure 5, there was a significant correlation between the SUN grading score and the grading value of each eye (rho: 0.748, *p* < 0.001).

## 4. Discussion

The SUN grading system is the gold standard for the assessment of anterior chamber inflammatory cells in uveitis. However, there are some limitations, including subjective clinical grading, low to moderate reproducibility for exact agreement in the grading of anterior chamber cells, and the effect of the biomicroscopic illumination system on the grading of anterior chamber cells. Therefore, improved systems for grading anterior chamber cells that are objective, quantitative, and reproducible are needed for clinical practice and clinical trials. In the present study, we developed an automated system to detect cellular spots in the anterior chamber using anterior segment OCT (CASIA 2), and we confirmed that the number of cellular spots in the anterior chamber was significantly correlated with the SUN grading scale in each eye. We calculated the approximated anterior chamber grading value with the logarithmic approximation curve between the SUN grading scale and the number of cellular spots in the anterior chamber, and LOOCV analysis demonstrated that the approximated anterior chamber grading value was significantly correlated with the SUN grading in each eye. 

For SUN grading systems of anterior chamber cells, examiners observe cells in a field size of 1 × 1 mm slit beam in the central part of the anterior chamber. Li and colleagues showed that the large and heavier cells could become trapped in the inferior part of the anterior chamber, suggesting that those cells might be missed by the slit-lamp biomicroscope [7,12]. In this study, we analyzed the cellular spots in the anterior chamber using the whole anterior chamber with four evenly distributed OCT images. Indeed, we confirmed that AS-OCT images of eyes having a grade of 0 according to the SUN grading showed cellular spots corresponding to the hyper-reflective spots in the anterior chamber, which was compatible with previous reports [11,14]. These findings indicate that multiple scan analysis or volume scan analysis using OCT images may be important for a more precise assessment of disease activity in anterior segment inflammation and in monitoring the response to the treatment in patients with uveitis.

The present study demonstrated that the number of cellular spots in the anterior chamber in an OCT image was positively correlated with the SUN clinical grade, and our automated system showed approximated anterior chamber grading values as the continuous measures of anterior chamber cells based on the logarithmic approximation curve. As shown in Figure 5, there was variability in the number of cellular spots detected in eyes with the same SUN grading. Furthermore, there was overlapping regarding the approximated anterior chamber grading value among eyes between grade 0 and 0.5+, between 0.5+ and 1+, and between 1+ and 2+ (Figure 5). These findings are in line with previous studies showing a tendency of a discrepancy, especially in low-grade inflammation of the anterior chamber [6,24]. These findings may be due to the nature of nonlinear and noncontinuous assessment of anterior chamber cells in the SUN grading system [4]. Kempen and colleagues have suggested that two ordinary changes in anterior chamber cell SUN grading would be a robust approach for diagnosing improvement and worsening in inflammatory status [6]. Thus, the continuous scale for anterior chamber cells presented in this study may be a more objective and quantitative outcome measure for the evaluation of anterior segment inflammation of uveitis.

Thus far, varying OCT devices, including time-domain OCT, spectral domain OCT, and swept-source OCT, have been used for studies to assess the anterior chamber cells in uveitis [7,8]. Recent studies have demonstrated the feasibility of AS-OCT for the quantification of anterior chamber cells in children with uveitis [25,26]. The accurate assessment of anterior chamber inflammation needs prolonged time to observe the anterior segment of uveitic eyes; however, younger children may be uncooperative for slit-lamp microscope examination due to the bright illumination of the slit-lamp beam [25,26]. SS-OCT used in this study has the advantage of high scanning speed, leading to a rapid scan time [15,16,27]. Faster AS-OCT image acquisition time by SS-OCT may be helpful for the assessment of anterior chamber inflammation in pediatric populations. 

Previous groups have shown that the number of hyperreflective spots segmented manually or automatically in the anterior segment has a significant correlation with the SUN grading score [11,12,14,15,16,27]. In the present study, since some clumps of hyperreflective spots due to cell-to-cell adhesion were seen in OCT images, we conducted the algorithm to segment the cellular spots in the anterior chamber using a single 5 × 5 pixel area instead of counting each hyperreflective spot for detection of cells in the anterior chamber in patients with uveitis. As shown in Figure 3, the number of cellular spots in the anterior chamber increased with an increasing SUN grading score. These findings are compatible with previous reports using anterior segment OCT [11,12,14,15,16,27]. However, our system was not able to distinguish inflammatory cells from floating pigment cells. Moreover, as shown in Figure 2 and Figure 3, eyes having a grade of 4+ according to SUN grading with severe fibrin formation and increased flare in the anterior chamber were automatically removed, indicating that the ability to distinguish inflammatory cells from floating materials, such as fibrin in uveitis eyes with severe anterior segment inflammation is limited. Recent studies have demonstrated the feasibility of artificial intelligence (AI)-based image analysis for anterior segment inflammation in uveitis [24], and the application of the AI system may be useful for the judgement of floating cells and materials in the anterior chamber in uveitic eyes.

The differentiation of cell types (neutrophils, lymphocytes, monocytes, and red blood cells) using AS-OCT devices remains a challenge. A previous study demonstrated the potential of AS-OCT to differentiate groups of inflammatory cells (polymorphonuclear pattern vs. mononuclear pattern) based on the reflectance distribution [13]. A recent study also has shown that the eyes of children with active juvenile idiopathic arthritis-associated uveitis and idiopathic panuveitis had a tendency towards populations of cells/particles with larger areas compared to the baseline of idiopathic chronic anterior uveitis [26]. Although our study did not evaluate the reflectance and size of each spot in each disease type, aqueous cell differentiation using AS-OCT may provide useful information for the diagnosis and approach to the treatment of uveitis. 

In the present study, we performed binarization for AS-OCT images. The algorithm identified and quantified hyperreflective signals that exceeded a certain threshold reflectance value in the anterior chamber. After this process, morphology transformation was performed to reduce noise interference. However, validation studies are needed to confirm whether there are no significant cellular spots on normal eyes using this automated algorithm. Unfortunately, the automated analysis of anterior chamber cells of normal eyes from healthy subjects was not approved by our institutional review board, and we were unable to perform this analysis. Therefore, it remains unknown to what extent our automated algorithm can detect signaling in the anterior chamber of normal eyes.

There are several limitations to this study. This study utilized a small sample size and was performed at a single institution. In addition, longitudinal studies are needed to confirm whether the present automated analysis of anterior chamber cells is useful for monitoring disease activity in patients with uveitis over follow-up. Further large-scale and multicenter studies are required for the validation of this method. In addition, since inflamed eyes tended to have brighter aqueous on AS-OCT due to a high concentration of inflammatory proteins in the anterior chamber, it is possible that increased aqueous reflectivity may not necessarily represent a higher number of white pixels representing cells in our system. Finally, although aqueous flare, as well as anterior chamber cells, are important inflammatory parameters for anterior segment inflammation of uveitis [4,10], the present study has not investigated the association between the number of cellular spots in the anterior chamber and aqueous flare level. Despite these limitations, our data have demonstrated that the clinical SUN grading score was significantly associated with the number of cellular spots in the anterior chamber using multiple scan images in uveitic eyes. Furthermore, we created continuous measures based on the SUN grading score through the logarithmic approximation curve between the clinical SUN grading score and the number of cellular spots in each eye. The current study highlights the potential of this automated method for objective, quantitative and rapid assessment of anterior chamber inflammation in clinical practices of uveitis. Further studies are needed to validate our findings and to establish a more precise assessment of anterior chamber inflammation in patients with uveitis.

## Figures and Tables

**Table 1:** Patient demographics.

Patients	31
Gender	
Men	21
Women	10
Age (yrs)	
Mean	55
Range	8–83
Diagnosis of uveitis	
VKH disease	4
Sarcoidosis	4
Behcet’s disease	2
CMV anterior uveitis	2
Herpetic keratouveitis	1
ARN	1
Unclassified	17

VKH: Vogt-Koyanagi-Harada, CMV: cytomegalovirus, ARN: acute retinal necrosis.

## Data Availability

Not applicable.
